# Factors Associated with Resilience of Adult Survivors Five Years after the 2008 Sichuan Earthquake in China

**DOI:** 10.1371/journal.pone.0121033

**Published:** 2015-03-26

**Authors:** Cuiping Ni, Meyrick Chum Ming Chow, Xiaolian Jiang, Sijian Li, Samantha Mei Che Pang

**Affiliations:** 1 Institute for Disaster Management and Reconstruction, Sichuan University-Hong Kong Polytechnic University, Chengdu, Sichuan Province, People’s Republic of China; 2 School of Nursing, The Hong Kong Polytechnic University, Hong Kong, People’s Republic of China; 3 Department of Nursing and Health Sciences, Tung Wah College, Hong Kong, People’s Republic of China; 4 West China School of Nursing/West China Hospital, Sichuan University, Chengdu, Sichuan Province, People’s Republic of China; Harvard Medical School, UNITED STATES

## Abstract

Given the paucity of quantitative empirical research on survivors’ resilience and its predictors in the context of long-term recovery after disasters, we examined how resilience predictors differed by gender among adult survivors five years after the Sichuan earthquake. This was a cross-sectional survey study of adult survivors (*N* = 495; aged 18–60) living in reconstructed communities five years into the recovery process after the Wenchuan earthquake. The instruments we used included assessments of sociodemographic characteristics and earthquake exposure level, the Connor-Davidson Resilience Scale, and the Social Support Rating Scale. Support-seeking behaviors emerged as a significant predictor of male survivors’ resilience, while subjective support and marital status were found to be predictors of female survivors’ resilience. Annual household income and chronic disease were predictors for both male and female groups. The findings of this study can be used in devising methods to boost survivors’ resilience by promoting their satisfaction with social support and their ability to obtain effective support. Additionally, the results suggest how to assist survivors who may have relatively poor resilience.

## Introduction

Natural disasters occur frequently all over the world and affect large populations. They can have extended impacts and recovering from them is a long-term process. Resilience has been shown to contribute to disaster victims’ recovery [1], and has been generally positively related to people’s mental health [2,3] and quality of life [4]. Given its clear importance, resilience has attracted considerable attention in both academic and popular discourse. Notably, resilience has been defined differently in reference to different groups (e.g., adolescent, adult, elderly people) and different contexts (e.g., trauma, academic performance) [5–8]. A generally accepted definition is the process of effectively adapting to and coping with adversity or stress by the use of protective resources [9,10]. Previous research [11–13] has also underscored the importance of the personal ability to “thrive under adversity” as an internal protective resource, noting that individuals almost always fall back on this ability in whatever adverse situation they encounter. As such, we defined resilience in this study as the personal ability to adapt to and cope with stressors, traumas, or adversity.

Because personal resilience can predict an individual’s adaptation to adversity and be used as an index in assessing the outcomes of trauma-related psychiatric disorders [12,13], it is crucial to know its status in the aftermath of a disaster and the sociodemographic characteristics that relate to low resilience in disaster survivors. Such knowledge would help in designing specific and effective interventions to boost resilience in this group. Post-disaster resilience is associated with positive outcomes, such as good mental health [14], well-being [15,16], and a lower incidence of posttraumatic stress disorder (PTSD) [17,18]. However, most previous studies have differed widely in their assessed resilience outcomes, nature of participants, and contexts, and as such, a comparison of their results would be inappropriate. Additionally, there have been a number of qualitative studies on post-disaster resilience, which focused primarily on small samples [19]. However, as useful as they are, these reports do not provide direct, quantitative measures of individuals’ resilience levels, limiting their generalization to other populations. Thus, there is a shortage of, and a need for, comparable quantitative empirical research on survivors’ resilience in the long-term post-disaster recovery process.

Previous studies have found gender to be associated with personal resilience. For example, Yu et.al [20] found men scored higher on resilience than women in a Chinese community. Additionally, Bonanno and Galea [21] reported that among participants who experienced the 9/11 World Trade Center attacks, men had a lower level of posttraumatic stress disorder (PTSD, an outcome related to resilience) than women. Tolin and Foa [22] further showed that regardless of study design or population, women tended to show higher levels of PTSD than men, suggesting lower resilience in women following trauma. However, despite the potential gender difference in resilience in the aftermath of a disaster, few studies have examined whether factors influencing resilience differ between genders.

Resilience has been found to be associated with social support in a variety of populations, such as disabled adolescents [4] and chronic disease patients [23]. However, little attention has been paid to the role of social support in disaster survivors’ resilience. Post-disaster victims usually receive various forms of external and explicit social support [24]. Social support is defined as assistance (tangible and intangible) or protection (shielding people from the adverse effects) exchanged between at least two individuals [25]. It has been categorized as two types, namely perceived availability of social support and actually received social support [26]. Social support has been identified as a significant factor moderating the adverse effects of sudden social and psychological trauma [24]. Although perceived social support has been reported to correlate with PTSD [27], it has not been reliably established whether social support affects resilience in post-disaster contexts. In many contexts, perceived social support (subjective social support) has been found to be more important to people than objective social support (received social support) [28,29]. However, objective social support is a basic form of support that is essential to natural disaster survivors. As such, there is also a need to explore the role of objective social support in resilience after experiencing a disaster. It has been suggested [30] that social support is more beneficial to women’s psychological health than to that of men. This warrants exploring the relationship between social support and resilience in different gender groups.

Numerous measures have been developed to quantify and assess resilience. One widely used scale, the Connor-Davidson Resilience Scale (CD-RISC) [11], was developed using the authors’ experience in clinical practice for treating PTSD and other mental disorders. In the CD-RISC, resilience is regarded as the personal ability to adapt to changes and cope with stressors [11], which makes it suitable for our study. Furthermore, the CD-RISC has proven to be a reliable scale in post-disaster populations [31]. Its validity and reliability have also been tested in Chinese adults and adolescent community populations [20,32]. Previous studies have pointed that there may exist gender differences in CD-RISC scores [2,20]. However, more research is needed to determine whether predictors of resilience after disaster differ in males versus females.

A serious earthquake, scaled at 8.0 Mw, occurred in Sichuan province, China, on May 12, 2008. This was a catastrophic event, which caused 69,142 deaths, with 17,551 people missing [33]. Beichuan County was the most seriously affected district, with 15,645 deaths, 26,916 people injured, 4,413 missing, and 142,000 rendered homeless in this district alone [34]. Utilizing numerous external support resources, as well as through their own efforts, many survivors have rebuilt their homes and reconstructed their lives. We believe that their experience of resilience would provide reliable empirical evidence to clarify the relationship between resilience, sociodemographic characteristics, and social support in different gender groups of disaster survivors.

The objectives of this study were to assess the resilience levels of adult survivors in reconstructed communities located in the most seriously damaged areas five years after the 2008 Sichuan earthquake, and to explore the predictors of resilience—including social-demographic characters, earthquake exposure level, and social support—for different gender groups.

## Methods

### Participants

The study was conducted among adult residents who had experienced the 2008 Wenchuan earthquake and its aftermath and lived in the four newly rebuilt resettlement residential communities. All participants of this study met the following inclusion criteria: ages ranged from 18 to 60 years old, participants had no cognitive impairments, and they were willing to sign the written consent form for voluntary participation.

### Measures

#### Sociodemographic characteristics and earthquake exposure level scale

The sociodemographic information that we assessed included age, gender, education level, ethnicity, marital status, chronic diseases, and earthquake exposure level (including whether participants had suffered injuries requiring hospitalized treatment and whether family members had died or gone missing in the earthquake).

#### Connor-Davidson Resilience Scale (CD-RISC; 25 items)

The CD-RISC has been used to assess earthquake survivors [32] and has been validated among Chinese community residents and adolescents [20,32]. It was translated into Chinese by Yu and Zhang [20], who failed to confirm the original five-factor structure derived from a U.S. sample, which consisted of the factors *personal competence*, *high standards*, *and tenacity; trust in one’s instincts*, *tolerance of negative affect*, *and the strengthening effects of stress; positive acceptance of change and secure relationships; control*; and *spiritual influences*. Instead, they found a three-factor structure in their Chinese sample, consisting of the factors *tenacity*, *strength*, and *optimism*. According to the CD-RISC manual, the total score of the entire scale is an indicator of the level of individual resilience. Items are rated on a five-point Likert scale, with answers ranging from 0 (“not true at all”) to 4 (“true nearly all the time”). The possible scores of the 25-item scale range from 0 to 100, with higher scores reflecting higher resilience. The factor structure and psychometric properties of the Chinese version of the CD-RISC were confirmed in a second study involving 2,914 Chinese adolescents living in Sichuan, China [32]. The Cronbach’s α reliability of the CD-RISC in this study is 0.875.

#### Social Support Rating Scale (SSRS)

Social support was evaluated using the Social Support Rating Scale (SSRS), which was designed by Xiao [35]. It consists of 10 items that fall into three dimensions: objective support, which refers to the degree of actual support an individual received (three items); subjective support, which refers to how individuals perceive their interpersonal support (four items); and support-seeking behavior, which refers to the individual’s pattern of behavior when seeking social support (three items). The subjective support dimension contains multiple sub-sections. One item in the dimension has five sub-items, each with a score ranging from 1 to 4 rating the level of perceived support from family members and relatives. The total score of the five sub-items constitutes the item score. For two of the three items in the objective support dimension, participants could choose multiple answers. Participants received 0 points for choosing one (no support resources) of the answers, and 1 point for each of the other 9 answers that pertained to them (receiving support from resources such as spouse, family members, government organizations, religious organizations). Thus, participants could receive a total score of 0 to 9 on these two items. The remaining items of the scale were forced-choice questions with 4-point scales ranging from 1 to 4. The total support score and scores for the subjective, objective, and support-seeking subscales ranged from 12 to 66, 8 to 32, 1 to 22, and 3 to 12, respectively. A higher score indicates greater social support. The scale is adapted to the Chinese culture and is widely used in the Chinese population [28]. The Cronbach’s α coefficient (a measure of internal consistency) of the whole scale was 0.91 in a previous study of earthquake survivors [36]. In another report on mental health workers who had no earthquake experience, Cronbach’s α for the total score and subscales ranged from 0.83 to 0.90, and the content validity and construct validities were both satisfactory [37]. The Cronbach’s α values for the whole scale and the subjective, objective, and support-seeking subscales in this study were 0.83, 0.66, 0.81, and 0.61, respectively.

### Data collection procedure and data analysis

This cross-sectional survey was conducted in July 2013. The investigators included the researchers and a research assistant who was trained by the researchers before the survey. They discussed each item and agreed on how to explain it if the participants did not understand. The investigators visited the participants’ homes and explained the purpose of the study, seeking voluntary participation. For participants who were illiterate, the investigators read the questions and answer options verbatim and recorded participants’ responses. The questionnaires were collected immediately after completion, and missing item responses were verified. Each questionnaire was digitally coded to avoid repeating or missing data, and the coded data were entered simultaneously into EpiData 3.1 by two researchers and then examined to correct missing or duplicate data. Statistical analyses were conducted using SPSS 19.0. Descriptive statistics and independent t-tests were used to assess the sociodemographic data. Pearson correlation coefficients were calculated to evaluate the relationship between CD-RISC scores and SRSS subscales. Multiple hierarchical linear regression analysis was performed to identify the predictors of CD-RISC scores and the contribution of each predictor to the explained variance. During the regression analysis, two dummy variables were created for the three education level variables where the primary school or illiteracy group data were the reference. The significance level was set at. 05 for all inferential statistical analyses in this study.

### Ethics

The study received ethical approval from the Hong Kong Polytechnic University Human Subjects Ethics Subcommittee. All participants were briefed about the purpose of the investigation and their right to not participate or to withdraw at any time. They were then asked to sign consent forms if they agreed to participate in the survey. During the entire research process, researchers ensured the confidentiality of participants’ answers to the questionnaires and only the research team members were allowed to access the raw materials and data.

## Results

### Participant characteristics

A total of 520 questionnaires were administered and 495 (95.2%) valid questionnaires were collected. Table 1 shows the characteristics of the participants.

**Table 1:** Sociodemographic characteristics of the participants.

				Gender	
Variables		N	%	Male (235)	Female (260)
Age	18–30	161	32.53	60	101
	31–45	222	44.85	112	110
	46–60	112	22.63	63	49
Marital status	Married	399	80.60	186	213
	Not married	96	19.39	49	47
Ethnic group	Han and others	232	46.87	125	138
	Qiang	263	53.13	110	122
Education level	Primary and lower	152	30.71	70	82
	Junior high school	214	43.23	104	110
	Senior high school and above	129	26.06	61	68
Chronic disease	Had	37	7.47	20	17
	Did not have	458	92.53	215	243
Annual household income (RMB)	<5000	207	41.82	101	106
	5000–20000	226	45.66	102	124
	20000–50000	45	9.09	23	22
	>50000	15	3.03	9	6
Family member dead/missing in earthquake	Family member dead/missing	150	30.30	60	90
	No dead/missing family members	345	69.70	175	170
Injured in the earthquake	Injured	84	16.97	38	46
	Not injured	411	83.03	197	214

### Resilience scores

Table 2 shows the resilience scores and compare them among different sociodemographic groups and by gender. Notably, male participants had higher resilience (Mean = 61.25, SD = 12.82) than did female participants (Mean = 58.00, SD = 12.00; *p* < 0.01). Female participants who had higher annual household incomes and had no chronic diseases had higher resilience than females with lower income (*p* < 0.001) or who had chronic disease (*p* < 0.01). Furthermore, male participants with higher annual household incomes also demonstrated higher resilience than males with lower income (*p* < 0.01). None of the other comparisons was significant (*p* > 0.05).

**Table 2:** Descriptive and univariate analysis of resilience by sociodemographic characteristics and gender.

Resilience		CD-RISC Mean (SD)	
		Male	Female
**Total**		61.25 (12.82)	58.00** (12.00)^a^
**Age**	18–30	62.37 (12.65)	57.83 (12.14)
	31–45	59.63 (12.69)	57.89 (11.95)
	46–60	63.05 (13.07)	58.57 (11.98)
**Marital status**	Married	61.04 (12.24)	57.31 (11.91)
	Not married	62.04 (14.93)	61.09 (11.98)
**Ethnic group**	Han and others	61.10 (11.84)	58.52 (12.32)
	Qiang	61.41 (13.91)	57.40 (11.62)
**Education level**	Primary and lower	60.29 (12.53)	57.63 (11.87)
	Junior high school	60.64 (12.54)	57.49 (12.91)
	Senior high school and higher	63.38 (13.58)	59.25 (13.54)
**Chronic disease**	Had	65.15 (12.60)	49.29** (10.91)
	Did not have	60.88 (12.81)	58.60 (11.84)
**Annual household income (RMB)**	<5000	57.81** (11.16)	54.44*** (12.03)
	5000–20000	62.66 (13.46)	60.08 (11.83)
	20000–50000	67.65 (13.42)	60.27 (9.16)
	>50000	67.44 (11.58)	68.66 (6.98)
**Family member dead/missing in earthquake**	Family member dead/missing	61.15 (12.54)	56.74 (11.36)
	No dead/missing family members	61.28 (12.95)	58.66 (12.28)
**Injured in the earthquake**	Injured	59.18 (13.01)	58.07 (11.83)
	Not injured	61.64 (12.78)	57.98 (12.70)

*p < 0.05

**p < 0.01

***p < 0.001

The comparison^a^ was between gender; other comparisons were within gender.

### Relationship between resilience and social support

Table 3 shows the social support scores and the Pearson correlations between resilience and social support. Subjective support, objective support, and support-seeking behaviors were significantly and positively correlated with resilience among female participants. The association between subjective support and resilience was the strongest of all of these relationships (r = 0.24, *p* < 0.01). However, among the male participants, only the support-seeking behaviors subscale was significantly associated with resilience (r = 0.19, *p* < 0.01).

**Table 3:** Means and standard deviations of social support and Pearson correlations between resilience and social support.

SRSS Subscale	Male		Female		Correlation with resilience	
	Mean	SD	Mean	SD	Male	Female
**Subjective**	22.70	4.72	23.44	4.83	0.12	0.24**
**Objective**	9.15	3.36	8.96	2.86	0.08	0.15*
**Support-seeking behaviors**	7.92	2.16	8.02	2.18	0.19**	0.19**

*p < 0.05

**p < 0.01

***p < 0.001

### Predictors of resilience

Table 4 presents the results of the hierarchical regression analyses of the contribution of sociodemographic characteristics and earthquake exposure variables (Step 1), and social support (Step 2) as predictors of resilience, separately for male and female participants. In the first step, for the male participants, annual household income (*β* = 0.25, *p* < 0.001) was the only significant predictor of resilience, explaining 9.1% of the total variance (*R* ^2^ = 9.1%, *p* < 0.05). However, for the female participants, significant predictors of resilience included not only annual household income (*β* = 0.23, *p* < 0.001), but also age (*β* = 0.19, *p* < 0.05), marital status (*β* = -0.16, *p* < 0.05), and chronic disease (*β* = -0.16, *p* < 0.05), which together contributed to 13.1% of the total variance (*R* ^2^ = 13.1%, *p* < 0.001). Female participants who were older, not married, or had no chronic diseases had significantly higher resilience levels than did females who were younger, married, or had chronic diseases. Annual household income was the strongest predictor of resilience for both the female and male participants.

**Table 4:** Multiple hierarchical linear regression analysis predicting resilience.

Independent Variables	Resilience (***β***)			
	Male		Female	
	Model 1	Model 2	Model 1	Model 2
Step 1: **Sociodemographic and earthquake exposure variables**				
Age	0.09	0.09	0.19*	0.14
Junior high school (ref. primary school or illiteracy)	0.01	-0.04	-0.02	0.01
Senior high school or higher (ref. primary school or illiteracy)	0.08	0.06	0.02	0.05
Marital status (married/not married)	-0.03	-0.07	-0.16*	-0.18**
Ethnic minority (Han and non-Qiang minorities/Qiang ethnic minority)	0.01	0.03	-0.02	0.01
Chronic diseases (yes/no)	-0.10	-0.15*	0.20**	0.18**
Annual household income	0.25***	0.25***	0.23***	0.19**
Family member dead/missing in earthquake (yes/no)	-0.01	-0.03	0.08	0.06
Injured in the earthquake (yes/no)	0.08	0.10	-0.03	-0.04
**Step 2: Social support**				
Objective support		-0.03		0.06
Subjective support		0.11		0.15*
Support-seeking behaviors		0.18*		0.05
*R* ^*2*^	0.09		0.13	
*F*	2.50*		4.14***	
Δ*R* ^*2*^		0.05		0.04
Δ*F*		4.36**		3.79*

*p < 0.05

**p < 0.01

***p < 0.001

In the second step, after controlling for sociodemographic and earthquake exposure variables, the male respondents who reported higher support-seeking behaviors also reported higher levels of resilience (*β* = 0.18, *p* < 0.05). Support-seeking behaviors explained an additional 5.1% of the variance in resilience (Δ*R* ^2^ = 5.1%, *p* < 0.01). In this model, annual household income was the strongest predictor (*β* = 0.25, *p* < 0.001), followed by support-seeking behaviors (*β* = 0.18, *p* < 0.05) and chronic disease (*β* = -0.15, *p* < 0.05). Overall, the full model explained 14.1% of the total variance in the male participants' resilience (*R* ^2^ = 14.1%, *p* < 0.01). The finding is surprising in that the male participants who had chronic diseases turned out to have higher resilience levels than did those who had no chronic diseases. After controlling for the same variables entered in the first step, we found that female respondents who reported higher subjective support (*β* = 0.15, *p* < 0.05) reported higher levels of resilience. Subjective support explained an additional 3.9% of the variance in the female participants' resilience (Δ*R* ^2^ = 3.9%, *p* < 0.05). In this model, annual household income was the strongest predictor (*β* = 0.19, *p* < 0.01), followed by chronic disease (*β* = 0.18, *p* < 0.01), marital status (*β* = -0.18, *p* < 0.01), and subjective social support (*β* = 0.15, *p* < 0.05). Overall, the full model explained 16.9% of the total variance in the female participants' resilience (*R* ^2^ = 16.9%, *p* < 0.05). Earthquake exposure variables did not emerge as significant predictors of resilience for both the male and female participants.

## Discussion

During the five-year period after the 2008 Wenchuan earthquake, stress-related syndromes may have been relieved once the critical response phase was over and survivors had begun to rebuild their lives. However, the effects of a disaster can last for years [38,39], and the development of resilience is a prolonged, dynamic process [40,41]. In the same context, some individuals may overcome adversity by developing toughness, while others may become overwhelmed [42]. Identifying what separates these individuals would be helpful in combating the lasting aftereffects of a disaster.

Our study set out to investigate the relationships between resilience, sociodemographic characteristics, and social support in adult earthquake survivors, with a specific focus on gender differences in predictors of resilience. The resilience level of survivors was lower than that found in a study by Yu and Zhang [20], who used the CD-RISC to assess a community sample of individuals in Guangdong Province who had not experienced the earthquake. Our study, like Yu and Zhang’s and Crabtree’s [43], found that male participants had higher levels of resilience than their female counterparts. For example, Yu and Zhang [20] found that male community residents of a Chinese city scored significantly higher on resilience than females. More relevant to the present study, Crabtree [43] reported that male survivors of a flood also scored significantly higher on resilience than their female counterparts. He argued that the observed gender difference might be attributable to different stressors faced by males and females and to females’ psychological dependence on males. Additionally, household responsibilities and lower perceived social support might also have contributed to the lower levels of resilience reported by women [44,45]. However, the findings of our study are inconsistent with those of studies conducted on adolescents [32,46]. In a study of Chinese adolescents’ resilience after an earthquake, Yu et al. [32] found that female adolescents reported significantly higher levels of resilience than did males in terms of the “spiritual influences” domain. Similarly, in a study of Australian adolescent refugees who had experienced migration and resettlement, Ziaian et.al [46] found that female adolescents scored significantly higher on resilience than male adolescents. The inconsistency between these two studies and the present study might be attributable to the different stressors that adults and adolescents encounter and the different external support resources available to them [43]. Furthermore, culture may also play a role in gender differences in resilience [43]. For example, in the Chinese culture, men show more tenacity than women, because men typically bear more of the burden of ensuring the family’s livelihood than women [47].

The separate regression analyses of resilience for the male and female participants revealed varying predictors of resilience by gender. While support-seeking behaviors were significant predicators for the male participants, marital status and subjective support were significant predictors for the female participants. Chronic disease and annual household income were the common significant predictors for both groups. The result regarding marital status for females—namely, that married female survivors scored significantly lower than unmarried female survivors on resilience—is perhaps due to stress resulting from bad marriages, the pressure of fostering children, and unanticipated increases in family financial burdens in married women, which may have affected their mental health and decreased their resilience. In contrast, unmarried female participants may have had lighter burdens after the earthquake and may have found it easier to recover from the disaster. In addition, after the earthquake, the majority of survivors had lost their lands and livelihoods, and the catastrophic damage to the local economy compelled many of the male survivors to leave their hometowns to find jobs elsewhere. In many cases, female participants might have been left behind by their spouses, which may have undermined their family coherence and further decreased their resilience. Marital status failed to emerge as a significant predictor of resilience among the male participants in this study. This may be due to cultural factors [43] and whether they are married or not, in Chinese culture men are always expected to show more tenacity than women when facing adversity [47].

The positive relationship between annual household income and resilience found in the current study is consistent with Kjellstrand and Harper [48], who reported that single mothers of middle-to-high annual income levels had higher resilience scores than their counterparts of low annual income levels. Bonanno and Galea [21] pointed out that survivors who experienced a loss of income in the aftermath of the 9/11 World Trade Center attacks were more likely to have several posttraumatic stress disorder symptoms and less likely to have resilience. Thus, the local economic downturn after Sichuan earthquake might have decreased survivors’ annual household income, which may in turn have affected their resilience.

With regard to social support, scores on all of the social support subscales were higher in this study than in Ke et al.’s [28] investigation of earthquake-hit areas eight months after the same earthquake. Possibly, participants recruited for this study—who were in the most seriously hit areas—received more social support than those sampled from less seriously damaged areas. Social support is traditionally divided into actual received social support (objective social support) and perceived social support (subjective social support). The current study found subjective social support to be a significant, positive predictor of resilience for the female participants, but not for the male participants. Objective social support was not a significant predictor for both gender groups. This is consistent with the findings of a previous study [49], which also found significant positive correlations between perceived (subjective) support and resilience. However, the observed non-significant relationship between objective social support and resilience does not mean that there is no need to assess objective support in the future. Ke et al. [28] suggested that the discrepancy between objective and subjective support scores could help us understand participants’ expectations about social support and thereby guide follow-up interventions.

This study found support-seeking behaviors to be a significant predictor of resilience for the male participants, but not for the female participants. Furthermore, male participants were found to be more likely to take initiative in seeking social support than their female counterparts. The male participants who took the initiative to talk about their troubles, seek emotional support, and elicit help, and were willing to attend collective activities, had significantly higher levels of resilience than did those who showed the opposite. This highlights the importance of improving survivors’ ability to express their troubles and obtain effective support and also suggests that survivors can improve their resilience levels by engaging in collective activities and building good relationships with people around them. Crucially, suitable interventions should be developed to improve satisfaction of social support and survivors’ desire to seek out support so as to improve their resilience.

Age emerged as a significant, positive predictor of female participants’ resilience when only sociodemographic variables were entered into the regression model, with older female survivors having higher levels of resilience. However, when the remaining variables were entered into the model, age was no longer a significant predictor of female participants’ resilience, indicating that other factors moderated the effect of age on resilience. Gucciardi et al. [50] also reported that there was no significant relationship between age and resilience in two age groups of Australian cricketers. This result, however, is inconsistent with the result of Yu et al. [32], which indicated that younger students had higher resilience than older students. It is possible that prior exposure to disasters is a better predictor of resilience than age. For example, Knight et al. [51] found that the degree of prior disaster experience, rather than chronological age, predicted post-disaster psychological functioning.

Our study found no significant relationship between education level and resilience among participants. This is inconsistent with findings from previous studies [47,52]. Frankenberg et al. [52] provided indirect evidence that male tsunami survivors who had more education had higher resilience five years after the disaster. Frankenberg et al. proposed that disaster survivors’ education levels might reflect the financial and social resources available to them during the post-disaster recovery period, which may affect their resilience outcomes. Li et al. [47] also reported that education may help boost one’s tolerance and overall mental health, which can in turn affect resilience. The observed absence of a significant relationship between survivors’ education and resilience levels might be attributable to the fact that the vast majority of the participants in this study had only a high school or lower education, which might have obscured the relationship due to homogeneity.

Like education, ethnicity also failed to emerge as a significant predictor for all the participants. This is inconsistent with findings from previous studies [11,47], which have revealed a significant relationship between ethnicity and resilience. The difference between the present study and previous research might be due to the homogenous culture, worldviews, and beliefs shared by the participants from different ethnic groups in this study, who had lived together and mixed with each other for several generations.

Having a chronic disease can increase the likelihood of PTSD and influence resilience [21], which was the case for the female participants in the current study. However, an interesting finding was that the male participants who had chronic diseases had higher resilience in this study. The male survivors who had chronic diseases may have felt lucky to remain alive after the catastrophic earthquake. Additionally, they may have gradually adapted to their illnesses before or after the earthquake and, with appropriate social support, successfully rebounded from adversity. They may not have been overwhelmed by their chronic disease and experiencing the disaster. In fact, they might have become tougher through coping with the challenges. In contrast, female survivors who had chronic diseases might have failed to adapt to their diseases and thus had lower resilience. Previous studies [53,54] have shown that women tend to report pain and pain anxiety more frequently than men during chronic disease, which may constitute a partial explanation for women’s low resilience. This result suggests that more attention should be paid to female survivors with chronic diseases when designing interventions for disaster survivors.

While adversity is a well-established factor relating to negative psychological outcomes [55], limited exposure to adversity could be beneficial in helping individuals develop resilience under the right circumstances after a period of time [42]. Scali [56] reported significant positive relationships between trauma exposure and resilience. However, the earthquake exposure variables (i.e., being injured in the earthquake and receiving hospitalized treatment, having a dead or missing family member due to the earthquake) were not significant predictors of resilience in this study. Of note is that our study was conducted five years after the earthquake. How long earthquake trauma and other trauma histories can influence individual resilience remains a topic for future study.

The above findings should be interpreted with caution in light of the following limitations. First, the study was conducted only in several reconstructed communities, which might limit the generalizability of its findings. Second, although a quantitative survey that was well equipped to reveal broad patterns about factors affecting survivors' resilience was used, it fell short of providing a nuanced understanding of how those factors influence survivors' resilience. Future studies taking a qualitative approach could contribute to a better understanding of how marital status, chronic disease, and different patterns of social support affect survivors’ resilience during the recovery process. Third, given its focus on the prediction of resilience by sociodemographic characteristics, earthquake exposure, and social support, the present study did not examine the possible effects of participants’ exposure to traumatic events over the 5 years since the disaster and their mental health status on resilience. Future studies should investigate the effects of these factors.

Despite these limitations, this study indicated that the predictors of resilience differed by gender, which offers new insights into the phenomenon of adaptive coping in different genders following disasters. It also revealed the factors that influence adult survivors’ ability to positively adapt to challenges following trauma.

## Conclusions

The general resilience levels of participants were relatively lower than were those found in similar studies. The findings of this study contribute to the empirical knowledge base that can be utilized to design post-disaster interventions that effectively deploy needed resources and services to improve adult survivors’ resilience. The fact that certain sociodemographic characteristics, such as marital status and chronic disease, and social support patterns played different roles in predicting resilience by gender should be taken into account when designing and implementing interventions to enhance survivors’ resilience. More support should be directed particularly toward married female survivors who suffer from chronic diseases.
